# Life-long dietary restrictions have negligible or damaging effects on late-life cognitive performance: A key role for genetics in outcomes

**DOI:** 10.1016/j.neurobiolaging.2022.07.004

**Published:** 2022-07-11

**Authors:** Andrew R. Ouellette, Niran Hadad, Andrew Deighan, Laura Robinson, Kristen O’Connell, Adam Freund, Gary A. Churchill, Catherine C. Kaczorowski

**Affiliations:** a The University of Maine, Graduate School of Biomedical Science and Engineering, Orono ME, USA; b The Jackson Laboratory, Bar Harbor ME, USA; c Calico Life Sciences LLC, San Francisco CA, USA

**Keywords:** Caloric restriction, Intermittent fasting, Cognition, Genetic diversity, Metabolism

## Abstract

Several studies report that caloric restriction (CR) or intermittent fasting (IF) can improve cognition, while others report limited or no cognitive benefits. Here, we compare the effects of 20% CR, 40% CR, 1-day IF, and 2-day IF feeding paradigms to ad libitum controls on Y-maze working memory (WM) and contextual fear memory (CFM) in a large population of Diversity Outbred mice that model the genetic diversity of humans. While CR and IF interventions improve lifespan, we observed no enhancement of working memory or CFM in mice on these feeding paradigms, and report 40% CR to be damaging to recall of CFM. Using Quantitative Trait Loci mapping, we identified the gene *Slc16a7* to be associated with CFM outcomes in aged mice on lifespan promoting feeding paradigms. Limited utility of dieting and fasting on memory in mice that recapitulate genetic diversity in the human population highlights the need for anti-aging therapeutics that promote cognitive function, with the neuronal monocarboxylate transporter MCT2 encoded by *Slc16a7* highlighted as novel target.

## 1. Introduction

Aging remains the greatest risk factor for the onset of dementia in the human population (Swerdlow, 2007). Given the current and projected increase of lifespan in the population, the risk of developing age-related disorders, such as dementia, increases in parallel (Christensen et al., 2009; Lunenfeld and Stratton, 2013). As such, the demand for interventions of cognitive decline is higher than ever. Dietary interventions, such as caloric restriction (CR) and intermittent fasting (IF), which are effective in extending lifespan (Mitchell et al., 2019; Pak et al., 2021; Weindruch et al., 1986), have been proposed as potential treatments for age-related cognitive disorders in both humans and animal models (Halagappa et al., 2007 Kishi et al., 2015; Kuhla et al., 2013; Leclerc et al., 2020; Witte et al., 2009). However, results from these studies are often conflicting; many studies report that CR improves cognition while others report no effect (Burger et al., 2010; Dal-Pan et al., 2011 Harder-Lauridsen et al., 2017; Martin et al., 2007; Pifferi et al., 2018; Scott et al., 2014). We hypothesize that the divergence of these results may, in part, be explained by a lack of genetic diversity in many animal studies which often use a single or a few inbred strains, in addition to the inability to control environmental factors in human studies that often disrupts expected outcomes (i.e., access to healthcare, education, income, population structure etc.) (Watts et al., 2011).

**Fig. 1.**
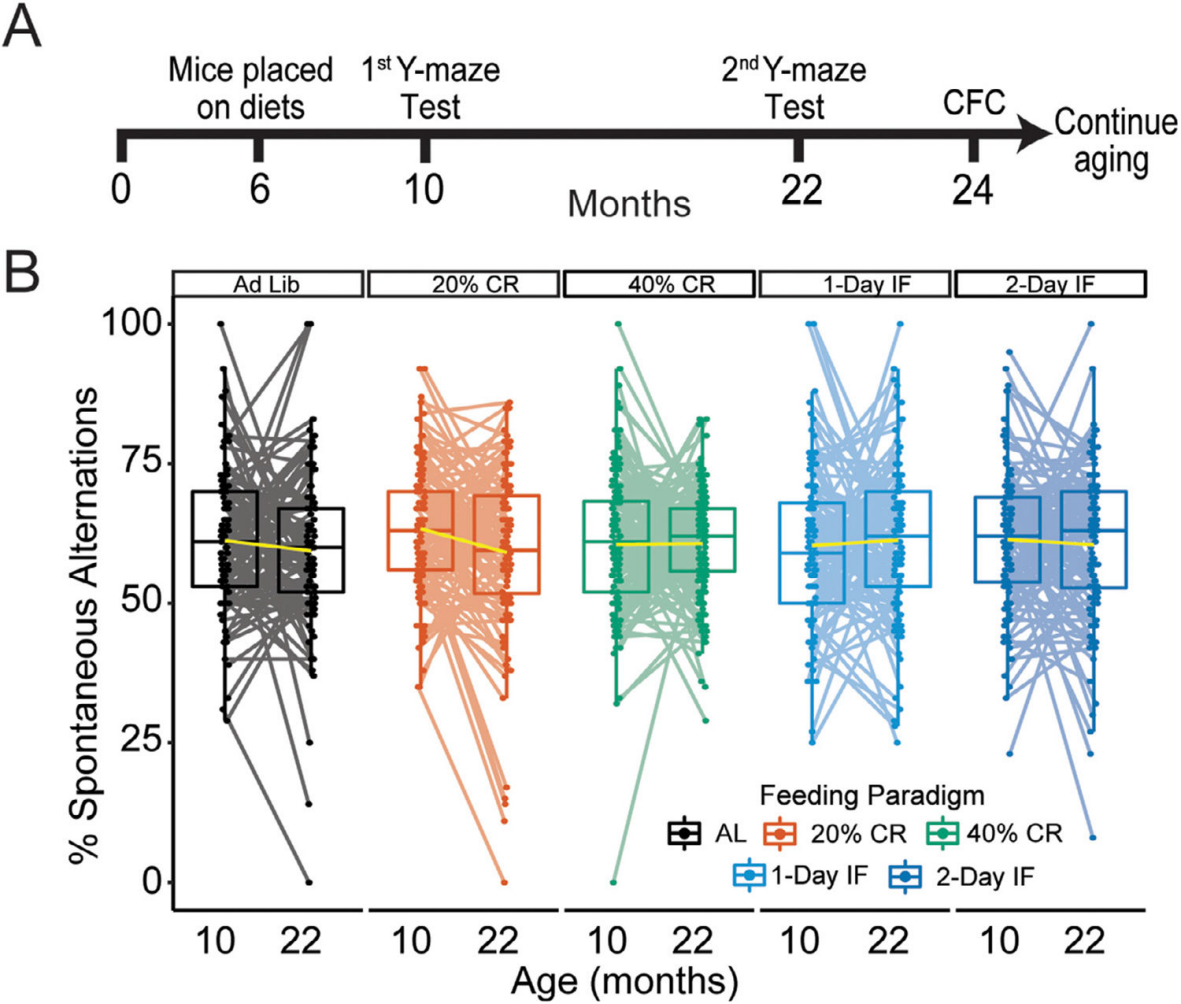
Caloric restriction and intermittent fasting paradigms have no effect on working memory in the Diversity outbred mouse population. (A) Overview of the experimental timeline; mice were longitudinally tested at 10 and 22 months on the Y-maze working memory task. After fear conditioning at 24 months mice were aged out to their maximum lifespan. (B) We observed no significant effects of diet on age related decline of working memory measured by % spontaneous alternations in the Y-maze working memory task from 10 months of age to 22. Each dot represents an individual mouse, yellow lines denote line of best fit between ages. Significance testing was performed using 2-way repeated measures ANOVA.

The Diversity Outbred (DO) mouse population offers the opportunity to study the effects of CR and IF on cognitive performance in a genetically diverse population of aged mice while still controlling environmental factors in a laboratory setting. The DO population is derived from 8 parental inbred lines, segregating for over 40 million single nucleotide polymorphisms that provide a great magnitude of genetic variation (Churchill et al., 2012). In this study, a population of 960 DO mice was randomly assigned to one of 5 feeding paradigms: *ad libitum* (AL) control, 20% or 40% CR, and 1 or 2-day IF. To assess the effect of aging, diet, and possible age x diet interactions on working memory, mice underwent longitudinal Y-maze working memory tests at 10 and 22-months of age (Fig 1A). Additionally, we assessed acquisition and recall of hippocampal-dependent spatial episodic memory using contextual fear conditioning (CFC) at 24 months of age. Previous studies have shown effects of long-term CR on survival in mice can be observed by 22–24 months of age, which is the approximate median lifespan of the DO population (Cameron et al., 2012; Mitchell, Sarah et al., 2019; Sun et al., 2013; Weindruch and Sohal, 1997), we therefore wanted to observe any potential changes in cognition at this age. Our results highlight the translational utility of the DO mouse population and the need for further mechanistic understanding of cognitive longevity to identify novel therapeutics.

## 2. Methods and materials

### 2.1. Animals

Female DO mice, obtained from the Jackson Laboratory, were used in this study (n = 960, J: DO, JAX stock number 009376, generations 24–28) previously described in (Churchill et al., 2012) as part of a longitudinal maximum lifespan study. Mice were housed in polycarbonate cages on ventilated racks providing 99.997% HEPA filtered air to each cage in a climate-controlled room (ambient air temperature of 22°C) under a standard 12:12 light-dark cycle (lights on at 0600 h). Mice were housed together in groups of 8 and provided with wood blocks and plastic tubes for enrichment. At 6 months of age mice were placed into one of the following 5 diet cohorts (n = 192 in each cohort). (1) *Ad libitum*; (2) 20% CR (2.75 g/mouse/d); (3) 40% CR (2.06 g/mouse/d); (4) 1 Day Fast (food removed Wednesday 15:00 and given Thursday 15:00); (5) 2 Day Fast (food removed Wednesday 15:00 and given Friday 15:00). Caloric restricted mice were provided food based on the number of mice in a given pen; mice in these pens were provided food at the same time. All mice were provided with 6% fat extruded grain (5K0G chow, LabDiet, St. Louis, MO, USA). All mouse experiments occurred at The Jackson Laboratory and were carried out in accordance with the standards of the Association for the Assessment and Accreditation of Laboratory Animal Care, as well as the recommendations of the National Institutes of Health Guide for the Care and Use of Laboratory Animals. The protocol was approved by the Institutional Animal Care and Use Committee at the Jackson Laboratory.

### 2.2. Genotyping

Tail tip samples were collected, and DNA extracted with the DNeasy Blood and Tissue Kit (Qiagen) from 954 animals (6 mice died before genotyping). Extracted DNA was genotyped on a 143,259-probe GigaMUGA array from the Illumina Infinium II platform (Morgan et al., 2016) by NeoGen Corp. Genotype quality was evaluated with the R/qtl2 package (Broman et al., 2019). All the raw genotype data was processed with a corrected physical map of the GigaMUGA array probes. After processing the genotype dataset contained 110,807 markers.

### 2.3. Y-maze working memory task

At 10 and again at 22 months of age, all mice were allowed to freely explore a Y-maze apparatus with 3 equally-sized arms for 8 minutes: arm length of 30 cm, arm lane width of 5–6 cm, wall height of 12–18 cm. External visual cues were removed using a curtain which surrounded the apparatus. Working memory was measured by % spontaneous alternations (Number of spontaneous alternations/Total arm Entries) between Y-maze arms. Recorded videos were analyzed using the ANY-maze behavioral tracking software (Stoelting Co, IL, USA). To limit the number of false positive arm entries we counted an arm entry when 99% of the mouse’s body (excluding tail) crossed into a new arm. Mice that did not make at least 6 total arm entries (the minimum number needed for at least 4 spontaneous alternations) were excluded from Y-maze data analysis.

### 2.4. Contextual fear conditioning

At 24 months of age all mice underwent CFC to assess the acquisition and recall of hippocampal-dependent memory (Neuner et al., 2015). On the first day of training, mice were placed in a training chamber and 4 foot-shocks (0.9 mA, 1 s) were delivered after a 150 second baseline period. Four post-shock intervals were defined as the 40 s following the end of each foot shock and the percentage of time spent freezing during each interval was determined using FreezeFrame software (Actimetrics Inc, IL, USA). The percentage of time spent freezing following the final shock was used as a measure of contextual fear acquisition across the panel. Twenty-four hours after training, mice were placed back into the training chamber and the percentage of time spent freezing throughout the entire 10-minutes test was measured as an index of contextual fear memory (CFM) recall; no shocks were delivered during the testing session. Mice were habituated in the testing room for 1 hour before testing/training on both day 1 and 2. Mice were fear conditioned on Monday/Tuesday to avoid testing during fasting periods.

### 2.5. Data analysis

Statistical analysis was performed using R 4.1.1. Survival: Mice that did not die from age-related causes (i.e., Missing or accidental death) were excluded from survival analysis. We fitted a logistic regression model with survival as a response variable and feeding paradigm as a fixed effect. This model accounts for clustering of mice into pens by using a nested random effect of pen within DO generation (given that any generation contains only a specific set of pens). The *p*-values from the mixed model were corrected for multiple testing using Benjamini-Hochberg correction. Y-maze: To test for differences in age related decline of working memory from 10 months to 22 months between diet cohorts we utilized a 2-way repeated measure analysis of variance; only mice that were in both age groups were analyzed. Contextual fear acquisition (CFA): Slope acquisition was derived from the coefficient of a linear model with the 4 post-shock values. To test for differences in CFC data between diets we utilized linear mixed modeling with the Lme4 R package (Bates et al., 2015). CFM: To test for a significant effect of diet on CFM we used linear mixed modeling with Lme4. In this model diet was treated as a fixed effect, test date as a random effect, and pen and generation as nested random effects. Post hoc *p*-values were derived from Tukey honestly significant difference (HSD) testing and corrected for multiple comparisons among the 5 diet cohorts using Holms correction for both CFA and CFM. Due to non-normal distribution, baseline percent freezing was log transformed in all analyses. Heritability: Heritability of cognitive phenotypes were calculated via the R/qtl2 “est_herit” function (Version 0.28) (Broman et al., 2019) by fitting a mixed-effects model controlling for feeding paradigm as a fixed effect and utilizing a genetic kinship matrix as a random effect.

### 2.6. QTL mapping

We performed Quantitative Trait Loci (QTL) mapping and single nucleotide polymorphism (SNP) association mapping with R using the qtl2 package (Version 0.28) (Broman et al., 2019). Log of the odds ratio (LOD) was derived from a linear mixed model and reported mapping statistic. The significance thresholds for QTL were calculated with the “scan1_perm” function in qtl2 with 10 0 0 permutations. For each permutation the genotype probabilities for each mouse are randomly assigned to different covariate and the maximum LOD score for the proceeding genome scan is recorded. To determine statistical significance of a QTL peak, a genome-wide *p*-value of 0.05 (95th percentile of recorded maximum LOD scores) from permutation testing was used. In genome scans, permutations and SNP association, we accounted for genetic similarity between mice using a kinship matrix with the leave-one-chromosome-out method. The founder allelic effect was identified using a regression of the phenotype on the founder genotype probabilities at each locus. For all CFM mapping, date of testing was used as an additive covariate to control for observed batch effects. To test for significant QTL across the entire DO population we used the 5 feeding paradigms as an interactive covariate. To test for significant QTL in mice on lifespan promoting diets, the DO population was separated to mice on 20% CR, 1-day IF and 2-day IF. These feeding paradigms were treated as a single covariate; therefore, no interactive mapping was used.

## 3. Results

### 3.1. Diversity outbred mice do not experience age-related working memory decline in a free exploring Y-maze task

The loss of working memory in aged humans and mice is well characterized in previous studies (Erickson and Barnes, 2003 Kirova et al., 2015; van Geldorp et al., 2015). However, the effect of CR and IF on working memory remains a point of contention in the literature. Here, we assessed working memory in young (10 month) and middle-aged (22 month) DO mice on AL, calorically restricted and intermittent fasted paradigms by measuring percent spontaneous alternation (%SA) in the Y-maze working memory task. We found that DO mice fed on the control AL paradigm exhibited a general trend towards an age-related decrease in %SA from 10 and 22 months, however, this effect was not statistically significant (*p* > 0.05) or affected by either CR or IF interventions in adult (10 months) or aged (22 months) DO mice (Fig. 1B). It is important to note that spatial cues were not provided to mice during free exploration of the Y-maze. In this configuration, the Y-maze task is not hippocampus-dependent and, therefore, not sensitive to age-related cognitive decline (Albani et al., 2014). As such, we are unable to determine if the absence of age-related decline is due to the lack of spatial references in the apparatus or true absence of age-related cognitive deficits (Lennartz, 2008).

### 3.2. Intermittent fasting confers no contextual fear memory benefits while severe caloric restriction is damaging in aged diversity outbred mice

**Fig. 2.**
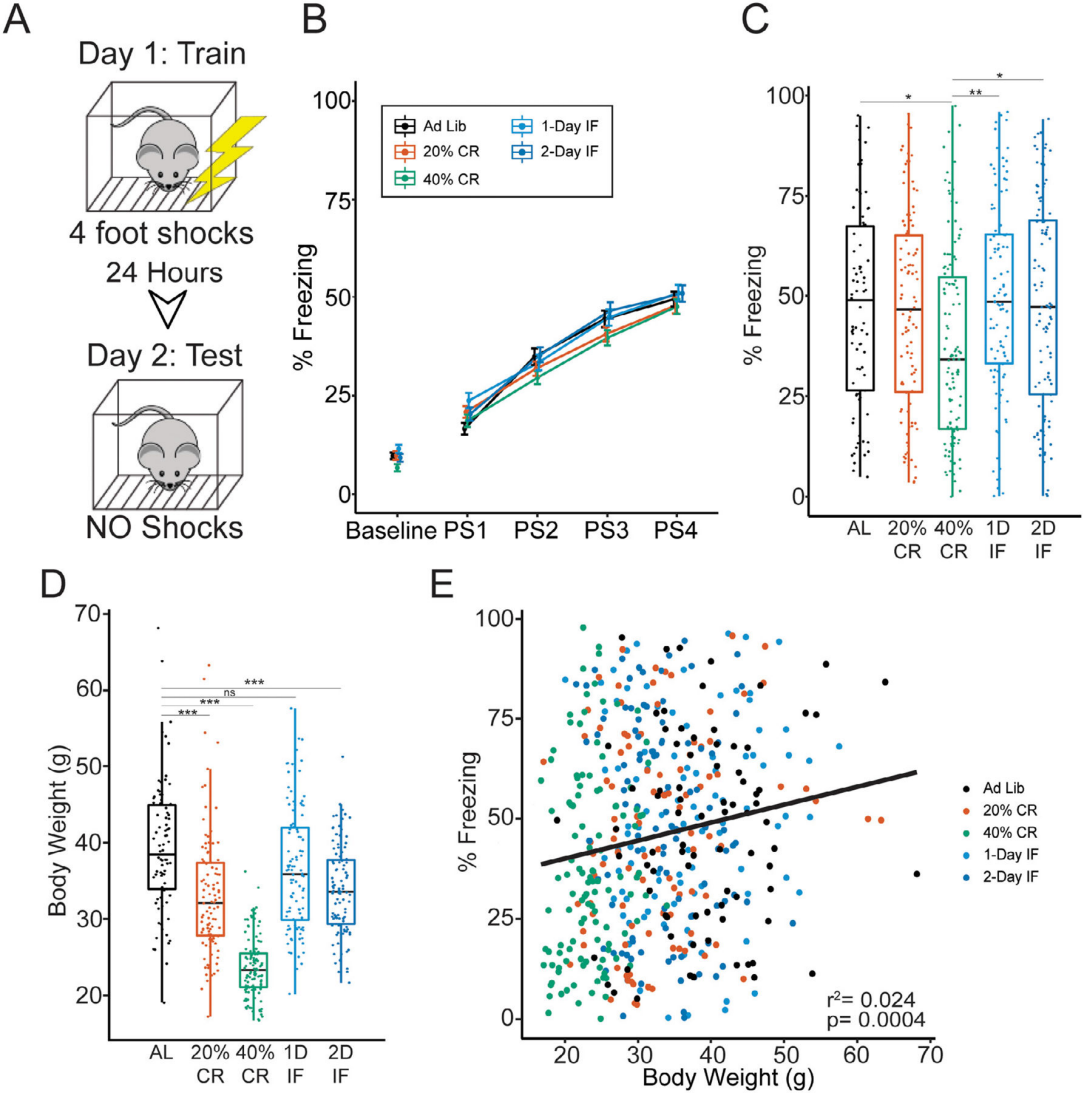
Contextual fear memory is impaired in diversity outbred mice on 40% caloric restriction compared to intermittent fasting and Ad Lib control groups. (A) CFC testing schematic; on day 1 mice were given 4 foot-shocks. Twenty-four hours later mice were placed in the same context without any shocks (B) % Freezing on day 1 of training before receiving shocks (baseline) and during each post-shock (PS) interval. Acquisition of CFC was comparable across all feeding paradigms. Data are expressed as mean ± SEM, significance was determined using 1-way repeated measures ANOVA. (C) Recall of Contextual Fear Memory (CFM) measured by total percent freezing of mice on day 2. Significant effect of diet on % Freezing was determined with a linear mixed model with test batch treated as a random effect. (D) Boxplot of body weights at 24 months of age across all feeding paradigms. A Post-hoc Tukey test was used to determine significant differences between diet groups; p-values were corrected for multiple comparisons using Holm correction (E) Scatter plot correlating CFM percent freezing and 24 month body weight across the entire DO population. ^ns^*p* > 0.05, * *p* < 0.05, ** *p* < 0.01 *** *p* < 0.001, n = 502. Boxplots encompass the 25th to 75th percentile with whiskers indicating 10th and 90th percentiles, median lines are indicated within each box.

The acquisition, consolidation and recall of spatial and contextual memories are known to be associated with hippocampal dysfunction in both animal models and humans, which is sensitive to the aging process (Park et al., 1996; Wimmer et al., 2012). To assess the effect of CR and IF interventions on the acquisition and recall of hippocampal-dependent memory relative to the AL controls in the DO mice, we performed CFC at 24 months. Acquisition of CFM was measured as the change in percent time spent freezing of the mice after each of 4 foot shocks on day 1 of training (Fig. 2A, top), previously characterized as a measurement of memory acquisition (Neuner et al., 2019). We found that mice acquire contextual fear (CFA, measured by slope acquisition and % freezing during post-shock 4) on day 1 of training comparably regardless of feeding paradigm, suggesting that neither long-term CR nor IF have a profound effect on the acquisition of CFC performance relative to AL (Fig. 2B, Fig. S1).

To assess the effects of CR and IF on CFM, we measured the total percent freezing of the mice during the day-2 testing period (Fig. 2A, bottom). To test for a main effect of feeding paradigm on CFM performance we used a linear mixed model that accounts for testing batch effects as a random effect in addition to nested pen density within different generations (F(4502) = 4.451, *p* < 0.01, Fig. 2C, Fig. S2). Post hoc analysis revealed no significant differences between AL controls and feeding interventions, except in the 40% CR cohort, which exhibited impaired CFM (Fig. 2C) (Tukey HSD, *p* = 0.03, 95% C.I. = AL [40.3, 54.9], 40% CR [30.3, 43.9]). We also observed that CFM in the 40% CR cohort was also significantly impaired compared to both IF interventions (Tukey HSD, *p* < 0.02, 95% C.I. = 40% CR [30.3, 43.9], 1D-IF [41.9, 55.8], 2D-IF [40.6, 54.5], Fig. 2C). These results suggest that, while CR and IF interventions reported herein replicate lifespan extension using percent survival at 24 months as a proxy measure (Table 1, (Chi-Square = 17.82 on 4 df, *p* = 0.001)), CR and IF do not promote cognitive performance in aged DO mice compared to AL feeding, and in the case of 40% CR, is damaging. Given the possibility that changes in the general activity of mice on 40% CR may have confounded our proxy for recall of CFM (Carter et al., 2009), we tested effects of CR and/or IF on general activity in the Y-maze task (measured by total distance traveled during exploration) at 22 months of age. We detected a main effect of feeding paradigm on Y-maze activity (F(4676) = 6.21, *p* < 0.0001) with a significant difference detected between AL controls and 40% CR (Tukey HSD, *p* < 0.001, 95% C.I. = AL[8.83, 11.0], 40% CR[11.87, 13.8]). Given that recording of Y-maze outcomes occurred 2 months prior to CFC, we also tested for effects of feeding paradigm on general baseline activity during the CFC task (measured as percent freezing on day 1 before receiving shocks). While we observed a main effect of feeding paradigm on baseline percent freezing ((F(4505) = 2.71, *p* < 0.05, Fig. 2B), there was not a significant difference in freezing between 40% CR and AL controls (Tukey HSD, *p* = 0.071, 95% C.I. = AL[1.57, 2.17], 40% CR[1.41, 1.97], data are log transformed). Additionally, we assessed post-shock CFA freezing across all shock intervals and found no significant effect of 40% CR on freezing (Fig. 2B). Since the 40% CR cohort shows comparable freezing during baseline and acquisition of CFM with AL controls and all other feeding paradigms, respectively, we interpret the decrease in freezing in 40% CR group during test of CFM recall to reflect a memory deficit as opposed to a general increase in activity.

In any feeding study changes in and correlation with body weight also need to be considered. As expected, we observed a significant effect of feeding paradigm in body weight at the median 24-month time point (F(4502) = 76.01, *p* < 0.0001), with 40% CR imparting the most severe reduction in body weight when compared to AL controls (Tukey HSD, *p* < 0.00 01, Fig. 2D). While individual changes in body weight contribute little variance to CFM outcomes (2.4% variance explained), we detected a significant positive correlation between these 2 traits (Fig. 2E), suggesting that metabolic outcomes altered by diet are coupled with late-life cognition.

The variance in CFA/CFM performance in this DO population is much higher than inbred mouse strains given the same conditioning, which can attributed to the population’s enhanced genetic diversity (Bolivar et al., 2001; Tuttle et al., 2018). Similar distribution in phenotypes in previous DO studies and have pointed to the significant impact of heritability (h^2^) on phenotypic outcomes (Keenan et al., 2020). To determine whether genetic variation can explain the high degree of variance in CFA/CFM, we calculated heritability for these traits using a linear mixed modeling approach with the R/qtl2 package (Broman et al., 2019). When accounting for variance between feeding paradigms, we calculate that 54.7% of the CFM variance can be explained by genetic diversity. This h^2^ estimate is much higher than that estimated for Y-maze %SA, providing further evidence that the lack of a working memory phenotype is likely due to environmental factors, such as visual cues (Table 2). We propose this high heritability not only makes large CFM variance permissible, but also encouraging, given that we expect individual genetic background to influence late-life cognitive outcomes. We next sought to determine if specific regions in the genome were associated with late-life cognitive outcomes and if any specific genes or variant could provide actionable therapeutic targets.

### 3.3. Slc16a7 is associated with contextual fear memory outcomes in mice on feeding paradigms that promote lifespan and preserve cognitive function

**Fig. 3.**
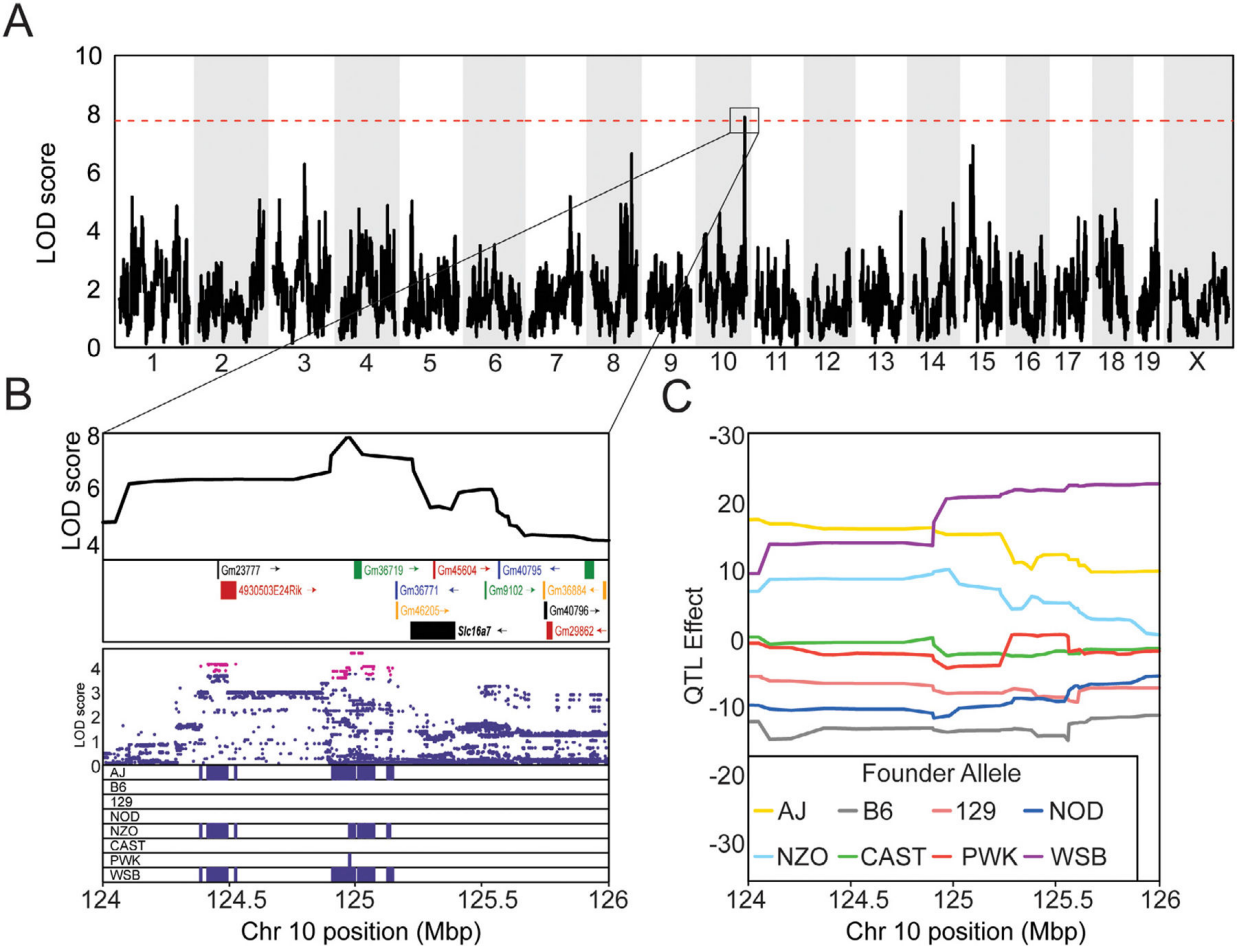
Slc16a7 is associated with variation long-term memory outcomes. (A) QTL map across the entire genome identifies a locus on chromosome 10 that significantly associates with changes in recall of CFM in aged DO mice. Significance score (LOD) is represented on the y-axis. The dashed red represents significance threshold from permutation testing (1000 permutations, *p*-value = 0.05) (B) (Top) Area under the significant QTL peak showing genes and gene modules within this region. (Bottom) SNP marker associations of the 8 DO founders labeled above. The x-axis shows the distribution along the chromosome in physical distance, SNPs colored in red fall within a LOD 1.5 drop of the peak marker. Minor allele frequency for SNPs significant SNPs is shown below. The x-axis is physical distance in Mb along the chromosome. (C) Haplotype effects of the 8 founders at the QTL region for CFM. The y-axis for the top panel is the effect coefficient.

While genetic background has been shown to modify cognitive decline in aged individuals, the exact genetic factors which underly these outcomes remain poorly understood. As such, we performed QTL mapping across our DO population to identify loci that are associated with worsened or enhanced cognitive outcomes in the face of dietary interventions. Mapping of Y-maze working memory, CFA or CFM with feeding paradigm as an additive and/or interactive covariate yielded no significant QTL peaks (data not shown) across the genome. The lack of a significant QTL peak in the highly heritable CFM data can, in part, result from of high degrees of freedom within our interactive QTL mapping (comparisons between 5 diet groups). In order to reduce the degrees of freedom within our QTL mapping, while still testing for genetic associations with behavior in mice from diet groups that were comparable in response to lifespan promoting feeding paradigms and cognitive outcomes, we mapped 20% CR, 1-day and 2-day IF cohorts as a single covariate. QTL mapping of late-life CFM outcomes in these mice revealed a significant peak near the end of chromosome 10 ranging from 124.2 to 125.3Mbp (alpha = 0.05, Fig. 3A). Within this locus we identified several gene modules, a Riken gene and the protein coding gene *Slc16a7* (Solute carrier family 16 member 7) (Fig. 3B, top). Additionally, we identified 256 SNPs within this region associated with changes in cognitive outcomes and are found in 3 of the DO founder strains (AJ, NZO, and WSB) (Fig. 3B Bottom). Four of these SNPs are upstream of GM23777 and 2 downstream; the remaining 250 SNPs are classified as intergenic. Based on previous research investigating the role of *Slc16a7* in metabolism and cognitive performance (Lev-Vachnish et al., 2019; Lu et al., 2015; Netzahualcoyotzi and Pellerin, 2020; Yu et al., 2021), we nominated this gene over identified gene modules. We next sought to determine if these SNPs were associated with enhanced or worsened late-life cognitive outcomes. Using a linear mixed model that accounts for kinship, we calculated QTL effects from each of the 8 DO founders along chromosome 10. We found that mice with AJ, NZO, and WSB alleles in the QTL peak region had higher late life cognitive function than mice with B6 or NOD alleles (Fig. 3C). These results indicate that variants within this region are associated with enhanced late-life cognitive performance in individuals on lifespan and cognitive health span promoting dietary interventions.

## 4. Discussion

Heritability estimates for age-related cognitive decline, including the onset of dementias such as Alzheimer’s disease are in the range of 58%–79% (Deary et al., 2012; Gatz et al., 2006; Harris and Deary, 2011; Ridge et al., 2013), suggesting that genetic background plays an integral role in regulating late-life cognitive outcomes. Studies that use traditional inbred rodent models of aging fail to capture the impact of genetic diversity on cognitive traits, resulting in a lack of translatability to the genetically diverse human population. Additionally, many of these studies reporting cognitive improvement in CR treated cohorts report only marginal improvement in memory tasks that assess only a single cognitive domain (Dhurandhar et al., 2013; Parikh et al., 2016; Wahl et al., 2018; Wu, Aiguo et al., 2003). Using a large panel of genetically diverse mice allowed us to robustly evaluate the effects of feeding interventions on cognitive performance across genetic backgrounds, enhancing the likelihood that results will generalize across diverse populations and species. Moreover, our results suggest genetic background may be a key factor contributing to conflicting findings in previous reports using model organisms of aging, given the complex nature of gene x diet interactions that are known to modify cognitive outcomes in genetically diverse models of dementia (Dunn et al., 2019; Tucker-Drob et al., 2013). While cross-sectional or longitudinal design is needed to fully assess the effects of CR and IF on age-related decline in CFM (McQuail et al., 2021), our study is the first of its kind to assess the effect of CR and IF on memory performance in a population of mice that actually models the genetic complexity of humans. We demonstrate the translational significance of the DO mouse population in the context of late-life cognitive deficits and highlight the variability that is expected in humans in response to pro-longevity interventions that is caused by increased genetic diversity. We show that a long-term 40% CR feeding regimen, whileeffective for extending lifespan, may be damaging for cognition across a diverse elderly population, suggesting that this method of dietary restriction may not stave off late-life cognitive deficits (or worse, enhance such deficits). Our future work with DO mice will include multiple cross-sectional time points in order to better understand cognitive longevity within a genetically diverse population. That said, our study is a critical first step towards understanding variation in the effect of dietary interventions on an outbred population of mice under a well-controlled experimental setting.

With our reported gene mapping results, we highlight the utility of DO mice in investigating the genetic factors associated with variation in the cognitive outcomes of aged mice. Genetic mapping using performance of CFM recall as a quantitative trait successfully identified *Slc16a7* as a potential mediator of cognitive outcomes in individuals undergoing lifespan promoting CR and IF interventions. *Slc16a7,* which codes the lactate and pyruvate transporter protein MCT2 (Monocarboxylate transporter 2), has previously been implicated in age-related cognitive dysfunction in rodents and humans via neuronal lactate transport (Lev-Vachnish et al., 2019; Lu et al., 2015; Netzahualcoyotzi and Pellerin, 2020; Yu et al., 2021). Lactate is an important energy source for de novo mRNA translation, a process critical for memory consolidation and recall (Descalzi et al., 2019), and MCT2 functions in hippocampal neurons to achieve this demand for energy (Netzahualcoyotzi and Pellerin, 2020). Conditional knockout of MCT2 in these neuronal populations has been shown to induce memory deficits in inbred B6 mice that parallel those observed in aged DO mice exhibiting CFM deficits (Netzahualcoyotzi and Pellerin, 2020). We propose that genetic variants in the region of *Slc16a7*, in conjunction with moderate CR and IF, contribute to individual variation in cognitive performance in aged DO mice. If there are intergenic variants affecting the expression of MCT2 in the DO population, it stands to reason that these changes in individual expression may impart observed cognitive enhancement or detriments. Future studies utilizing genetically diverse mouse populations will investigate the role of these variants in altering the expression of MCT2 and the modification of cognitive outcomes, and whether/if they are altering the function of MCT2 proteins or their level of expression in hippocampal neurons. Given the prior evidence for *Slc16a7* as a mediator of cognitive outcome, we nominate it as the gene of interest from our QTL mapping rather than identified gene modules. However, because significant variants that associate with enhanced cognitive outcomes are found within and around other gene modules and Riken genes, further investigation into GM36719, GM23777, and 4930503E24Rik is warranted and may yield new therapeutic targets and knowledge of late-life cognitive outcomes. Identification of *Slc16a7* in conjunction with the high heritability of late-life CFM demonstrates that the DO mouse population can effectively model the aging process across a genetically diverse population and shows that this process can be linked to previously confirmed genetic mechanisms.

While CR and IF are highly replicable and well established interventions of health-span and lifespan across multiple species ranging from worms to humans (Catterson et al., 2018; Lakowski and Hekimi, 1998; Mattison et al., 2017; Mitchell et al., 2019; Most et al., 2017; Rogina and Helfand, 2004) and in our own data, their efficacy to promote late-life cognitive performance has been mixed; the reason for this remains largely unexplored. There are many factors that may interact with CR and modify its efficacy as an intervention of cognitive decline, including genetic background and sex. Previous studies have shown that CR is more effective at promoting lifespan in certain inbred mouse strains, while less effective in others (Liao et al., 2013; Liao et al., 2010; Mitchell et al., 2016; Wilkie et al., 2020), suggesting that certain individuals may be predisposed to effectively utilizing CR strategies. Our work has shown that these methods are not effective at promoting late-life cognitive function on a population level. Additionally, previous studies have shown that sex modifies the effect of dietary restrictions on maximum lifespan and cognition (Kane et al., 2018; Mitchell et al., 2016; Wu et al., 2003; Zajitschek et al., 2013). It is therefore important to consider gene x sex interactions when evaluating the efficacy of CRs on both lifespan and cognitive aging. Due to animal health and attrition concerns stemming from previously observed aggression in DO males, only females were used in this study. Future work will need to include both males and females in order to determine gene x sex interactions that may affect the efficacy of dietary restrictions.

We also need to consider the role of survival bias in the interpretation of our results given that there is an increase in survival in the 40% CR cohort compared to AL controls. The possibility that the genetic factors potentially associated with decreased survival in the AL group also enhanced their cognitive abilities is a valid consideration. However, since DO mice were randomized across feeding cohorts, we expect an equal distribution of genotypes within each cohort. Furthermore, if a relationship between survival and cognition existed and induced a survival bias, we would (1) Expect to see it in other pro-longevity paradigms (1- and 2-day IF and 20% CR); and (2) Pro-longevity paradigms would result in improved cognitive outcomes. Therefore, if there was a selection bias against late-life poor cognitive performers in the AL cohort, we would expect to see relatively more of these poor performer mice in enhanced survival cohorts and a reduction in cognitive outcomes in the 20% CR, 1-day and 2-day IF cohorts; this is not the case.

## 5. Conclusion

DO mice have been shown to be an effective tool in targeting genetic mechanisms of aging that reliably translate to humans (Church et al., 2015; French et al., 2015; Ouellette et al., 2020; Recla et al., 2014; Tuttle et al., 2018). We, therefore, highlight the need to investigate alternative therapeutics of not only lifespan, but also cognitive health-span, given that CR and IF are proving to fall short of desired outcomes. In order to develop more effective therapeutics, we need to develop a better understanding of the genetic mechanisms of cognitive longevity; our future work will continue utilizing DO mice to do just this. By harnessing the complex genetic backgrounds of DO mice, we will investigate how genetic mechanisms regulate not only cognitive outcomes, but also molecular profiles (RNA expression and DNA methylation) and dendritic spine morphology that we and others showed associates with cognitive resilience in normal and AD aging (Kasai et al., 2010; Walker et al., 2021). These studies will provide insights into new genetic targets for potential therapeutics to rescue cognitive deficits and dementia.

## Supplementary Material

Supplementary

## Figures and Tables

**Table 1:** Caloric restriction and intermittent fasting paradigms increase percent survival at 24 months of age.

Feeding paradigm	% Survived to 24 mo	Total n included	*p*-value compared to ad lib
Ad lib	46.3	175	NA
20% CR	59.3	172	a
40% CR	68.0	169	^c^
1-d IF	59.0	173	^a^
2-d IF	60.5	172	b

Proportion of mice that survived until 24 months within each diet cohort. Mice that died from non-natural causes (i.e., missing animals, mishandling and non-endpoint euthanasia) were excluded from survival analysis. We observed that all diet interventions conferred increased survival compared to ad lib controls, 40% CR confers the greatest % survival [χ^2^ (4, n = 861) = 17.4, *p* = 0.002]. Post hoc testing was performed with multiple chi-squared tests comparing diet cohorts to ad lib controls, Holm multiple testing correction was used to correct multiple testing error.

a *p* < 0.05.

b *p* < 0.01.

c *p* < 0.001.

**Table 2:** Heritability of cognitive phenotypes within the DO population.

Trait	Heritability (h^2^)
Contextual fear acquisition (PS4)	20.0%
Contextual fear acquisition (Slope)	< 1%
Recall of contextual fear memory	54.7%
Y-maze % spontaneous alternation (10 mo)	5.4%
Y-maze % spontaneous alternation (22 mo)	2.6%

h^2^ is presented as a percent of the variation in a phenotype that can be explained by differences in genetic background across the DO population. A higher percentage is indicative of a greater percentage of variability explained by genetics.

## Abbreviations:

CR: caloric restriction
IF: intermittent fasting
DO: diversity outbred
%SA: percent spontaneous alternations
CFM: contextual fear memory
CFA: contextual fear acquisition
LOD: log of odds
ANOVA: analysis of variance
SEM: standard error of mean

## References

[R1] Albani SH, McHail DG, Dumas TC, 2014. Developmental studies of the hippocampus and hippocampal-dependent behaviors: insights from interdisci-plinary studies and tips for new investigators. Neurosci. Biobehav. Rev 43, 183–190. 24769291 10.1016/j.neubiorev.2014.04.009 PMC4939092

[R2] Bates D, Mächler M, Bolker B, Walker S, 2015. Fitting linear mixed-effects models using lme4. J. Stat. Softw 1 (1), 2015.

[R3] Bolivar VJ, Pooler O, Flaherty L, 2001. Inbred strain variation in contextual and cued fear conditioning behavior. Mamm. Genome 12 (8), 651–656. 11471061 10.1007/s003350020039

[R4] Broman KW, Gatti DM, Simecek P, Furlotte NA, Prins P, Sen Ś, Yandell BS, Churchill GA, 2019. R/qtl2: Software for mapping quantitative trait loci with high-dimensional data and multiparent populations. Genetics 211 (2), 495–502. 30591514 10.1534/genetics.118.301595 PMC6366910

[R5] Burger JM, Buechel SD, Kawecki TJ, 2010. Dietary restriction affects lifespan but not cognitive aging in Drosophila melanogaster. Aging Cell 9 (3), 327–335. 20156204 10.1111/j.1474-9726.2010.00560.x

[R6] Cameron KM, Miwa S, Walker C, von Zglinicki T, 2012. Male mice retain a metabolic memory of improved glucose tolerance induced during adult onset, short-term dietary restriction. Longev. Healthspan 1 (1), 3. 24764509 10.1186/2046-2395-1-3 PMC3886267

[R7] Carter CS, Leeuwenburgh C, Daniels M, Foster TC, 2009. Influence of calorie restriction on measures of age-related cognitive decline: role of increased physical activity. J. Gerontol. A Biol. Sci. Med. Sci 64 (8), 850–859. 19420296 10.1093/gerona/glp060 PMC2709546

[R8] Catterson JH, Khericha M, Dyson MC, Vincent AJ, Callard R, Haveron SM, Rajasingam A, Ahmad M, Partridge L, 2018. Short-term, intermittent fasting induces long-lasting gut health and tor-independent lifespan extension. Curr. Biol 28 (11), 1714–1724 e1714.. 29779873 10.1016/j.cub.2018.04.015 PMC5988561

[R9] Christensen K, Doblhammer G, Rau R, Vaupel JW, 2009. Ageing populations: the challenges ahead. Lancet 374 (9696), 1196–1208. 19801098 10.1016/S0140-6736(09)61460-4 PMC2810516

[R10] Church RJ, Gatti DM, Urban TJ, Long N, Yang X, Shi Q, Eaddy JS, Mosedale M, Ballard S, Churchill GA, Navarro V, Watkins PB, Threadgill DW, Harrill AH, 2015. Sensitivity to hepatotoxicity due to epigallocatechin gallate is affected by genetic background in diversity outbred mice. Food Chem. Toxicol 76, 19–26. 25446466 10.1016/j.fct.2014.11.008 PMC4324012

[R11] Churchill GA, Gatti DM, Munger SC, Svenson KL, 2012. The diversity outbred mouse population. Mamm. Genome 23 (9-10), 713–718. 22892839 10.1007/s00335-012-9414-2 PMC3524832

[R12] Dal-Pan A, Pifferi F, Marchal J, Picq JL, Aujard F, 2011. Cognitive performances are selectively enhanced during chronic caloric restriction or resveratrol supplementation in a primate. PloS One 6 (1), e16581. 21304942 10.1371/journal.pone.0016581 PMC3031601

[R13] Deary IJ, Yang J, Davies G, Harris SE, Tenesa A, Liewald D, Luciano M, Lopez LM, Gow AJ, Corley J, Redmond P, Fox HC, Rowe SJ, Haggarty P, McNeill G, Goddard ME, Porteous DJ, Whalley LJ, Starr JM, Visscher PM, 2012. Genetic contributions to stability and change in intelligence from childhood to old age. Nature 482 (7384), 212–215. 22258510 10.1038/nature10781

[R14] Descalzi G, Gao V, Steinman MQ, Suzuki A, Alberini CM, 2019. Lactate from astrocytes fuels learning-induced mRNA translation in excitatory and inhibitory neurons. Commun. Biol 2 (1), 247. 31286064 10.1038/s42003-019-0495-2 PMC6606643

[R15] Dhurandhar EJ, Allison DB, van Groen T, Kadish I, 2013. Hunger in the absence of caloric restriction improves cognition and attenuates Alzheimer’s disease pathology in a mouse model. PloS One 8 (4), e60437. 23565247 10.1371/journal.pone.0060437 PMC3614512

[R16] Dunn AR, O’Connell KMS, Kaczorowski CC, , 2019. Gene-by-environment interactions in Alzheimer’s disease and Parkinson’s disease. Neurosci. Biobehav. Rev 103, 73–80. doi:10.1016/j.neubiorev.2019.06.018. 31207254 PMC6700747

[R17] Erickson CA, Barnes CA, 2003. The neurobiology of memory changes in normal aging. Exp. Gerontol 38 (1-2), 61–69. 12543262 10.1016/s0531-5565(02)00160-2

[R18] French JE, Gatti DM, Morgan DL, Kissling GE, Shockley KR, Knudsen GA, Shepard KG, Price HC, King D, Witt KL, Pedersen LC, Munger SC, Svenson KL, Churchill GA, 2015. Diversity outbred mice identify population-based exposure thresholds and genetic factors that influence benzene-induced genotoxicity. Environ. Health Perspectives 123 (3), 237–245. 10.1289/ehp.1408202 PMC4348743 25376053

[R19] Gatz M, Reynolds CA, Fratiglioni L, Johansson B, Mortimer JA, Berg S, Fiske A, Pedersen NL, 2006. Role of genes and environments for explaining alzheimer disease. Arch. Gen. Psychiatry 63 (2), 168–174. 16461860 10.1001/archpsyc.63.2.168

[R20] Halagappa VK, Guo Z, Pearson M, Matsuoka Y, Cutler RG, Laferla FM, Mattson MP, 2007. Intermittent fasting and caloric restriction ameliorate age-related behavioral deficits in the triple-transgenic mouse model of Alzheimer’s disease. Neurobiol. Dis 26 (1), 212–220. 17306982 10.1016/j.nbd.2006.12.019

[R21] Harder-Lauridsen NM, Nielsen ST, Mann SP, Lyngbæk MP, Benatti FB, Langkilde AR, Law I, Wedell-Neergaard AS, Thomsen C, Møller K, Karstoft K, Pedersen BK, Krogh-Madsen R, 2017. The effect of alternate-day caloric restriction on the metabolic consequences of 8 days of bed rest in healthy lean men: a randomized trial. J. App. Physiol 122 (2), 230–241. 10.1152/japplphysiol.00846.2016 27881670

[R22] Harris SE, Deary IJ, 2011. The genetics of cognitive ability and cognitive ageing in healthy older people. Trends Cogn. Sci 15 (9), 388–394. 21840749 10.1016/j.tics.2011.07.004

[R23] Kane AE, Sinclair DA, Mitchell JR, Mitchell SJ, 2018. Sex differences in the response to dietary restriction in rodents. Curr. Opin. Physiol 6, 28–34. 31231711 10.1016/j.cophys.2018.03.008 PMC6588196

[R24] Kasai H, Fukuda M, Watanabe S, Hayashi-Takagi A, Noguchi J, 2010. Structural dynamics of dendritic spines in memory and cognition. Trends Neurosci. 33 (3), 121–129. 20138375 10.1016/j.tins.2010.01.001

[R25] Keenan BT, Galante RJ, Lian J, Simecek P, Gatti DM, Zhang L, Lim DC, Svenson KL, Churchill GA, Pack AI, 2020. High-throughput sleep phenotyping produces robust and heritable traits in diversity outbred mice and their founder strains. Sleep 43 (5). 10.1093/sleep/zsz278 PMC7215270 32074270

[R26] Kirova AM, Bays RB, Lagalwar S, 2015. Working memory and executive function decline across normal aging, mild cognitive impairment, and Alzheimer’s disease. BioMed Res. Int 2015, 748212. 26550575 10.1155/2015/748212 PMC4624908

[R27] Kishi T, Hirooka Y, Nagayama T, Isegawa K, Katsuki M, Takesue K, Sunagawa K, 2015. Calorie restriction improves cognitive decline via up-regulation of brain-derived neurotrophic factor: tropomyosin-related kinase B in hippocampus of obesity-induced hypertensive rats. Int. Heart J 56 (1), 110–115. 25503654 10.1536/ihj.14-168

[R28] Kuhla A, Lange S, Holzmann C, Maass F, Petersen J, Vollmar B, Wree A, 2013. Lifelong caloric restriction increases working memory in mice. PloS one 8 (7), e68778. 23874758 10.1371/journal.pone.0068778 PMC3707851

[R29] Lakowski B, Hekimi S, 1998. The genetics of caloric restriction in Caenorhabditis elegans. In: Proceedings of the National Academy of Sciences of the United States of America, 95, pp. 13091–13096. 9789046 10.1073/pnas.95.22.13091 PMC23719

[R30] Leclerc E, Trevizol AP, Grigolon RB, Subramaniapillai M, McIntyre RS, Brietzke E, Mansur RB, 2020. The effect of caloric restriction on working memory in healthy non-obese adults. CNS Spectr. 25 (1), 2–8. 30968820 10.1017/S1092852918001566

[R31] Lennartz RC, 2008. The role of extramaze cues in spontaneous alternation in a plus-maze. Learn. Behav 36 (2), 138–144. 18543713 10.3758/lb.36.2.138

[R32] Lev-Vachnish Y, Cadury S, Rotter-Maskowitz A, Feldman N, Roichman A, Illouz T, Varvak A, Nicola R, Madar R, Okun E, 2019. L-lactate promotes adult hippocampal neurogenesis. Front. Neurosci 13. 10.3389/fnins.2019.00403 PMC6542996 31178678

[R33] Liao C-Y, Johnson TE, Nelson JF, 2013. Genetic variation in responses to dietary restriction–an unbiased tool for hypothesis testing. Exp. Gerontol 48 (10), 1025–1029. 23562825 10.1016/j.exger.2013.03.010 PMC3759609

[R34] Liao C-Y, Rikke BA, Johnson TE, Diaz V, Nelson JF, 2010. Genetic variation in the murine lifespan response to dietary restriction: from life extension to life shortening. Aging Cell 9 (1), 92–95. 19878144 10.1111/j.1474-9726.2009.00533.x PMC3476836

[R35] Lu W, Huang J, Sun S, Huang S, Gan S, Xu J, Yang M, Xu S, Jiang X, 2015. Changes in lactate content and monocarboxylate transporter 2 expression in A*β*25-35-treated rat model of Alzheimer’s disease. Neurol. Sci 36 (6), 871–876. 25647291 10.1007/s10072-015-2087-3

[R36] Lunenfeld B, Stratton P., 2013. The clinical consequences of an ageing world and preventive strategies. Best practice & research. Clin. Obstet. Gynaecol 27 (5), 643–659. 10.1016/j.bpobgyn.2013.02.005 PMC3776003 23541823

[R37] Martin CK, Anton SD, Han H, York-Crowe E, Redman LM, Ravussin E, Williamson DA., 2007. Examination of cognitive function during six months of calorie restriction: results of a randomized controlled trial. Rejuvenation Res. 10 (2), 179–190. 17518698 10.1089/rej.2006.0502 PMC2664681

[R38] Mattison JA, Colman RJ, Beasley TM, Allison DB, Kemnitz JW, Roth GS, Ingram DK, Weindruch R, de Cabo R, Anderson RM, 2017. Caloric restriction improves health and survival of rhesus monkeys. Nat. Commun 8 (1), 14063. 28094793 10.1038/ncomms14063 PMC5247583

[R39] McQuail JA, Dunn AR, Stern Y, Barnes CA, Kempermann G, Rapp PR, Kaczorowski CC, Foster TC, 2021. Cognitive Reserve in Model Systems for Mechanistic Discovery: The Importance of Longitudinal Studies. Frontiers in Aging Neuroscience 12. 10.3389/fnagi.2020.607685 PMC7859530 33551788

[R40] Mitchell SJ, Bernier M, Mattison JA, Aon MA, Kaiser TA, Anson RM, Ikeno Y, Anderson RM, Ingram DK, de Cabo R, 2019. Daily fasting improves health and survival in male mice independent of diet composition and calories. Cell Metab. 29 (1), 221–228 e223.. 30197301 10.1016/j.cmet.2018.08.011 PMC6326845

[R41] Mitchell SJ, Madrigal-Matute J, Scheibye-Knudsen M, Fang E, Aon M, Gonzalez-Reyes JA, Cortassa S, Kaushik S, Gonzalez-Freire M, Patel B, Wahl D, Ali A, Calvo-Rubio M, Burón MI, Guiterrez V, Ward TM, Palacios HH, Cai H, Frederick DW, Hine C, Broeskamp F, Habering L, Dawson J, Beasley TM, Wan J, Ikeno Y, Hubbard G, Becker KG, Zhang Y, Bohr VA, Longo DL, Navas P, Ferrucci L, Sinclair DA, Cohen P, Egan JM, Mitchell JR, Baur JA, Allison DB, Anson RM, Villalba JM, Madeo F, Cuervo AM, Pearson KJ, Ingram DK, Bernier M, de Cabo R, 2016. Effects of sex, strain, and energy intake on hallmarks of aging in mice. Cell Metab. 23 (6), 1093–1112. 27304509 10.1016/j.cmet.2016.05.027 PMC4911707

[R42] Morgan AP, Fu C-P, Kao C-Y, Welsh CE, Didion JP, Yadgary L, Hyacinth L, Ferris MT, Bell TA, Miller DR, Giusti-Rodriguez P, Nonneman RJ, Cook KD, Whitmire JK, Gralinski LE, Keller M, Attie AD, Churchill GA, Petkov P, Sullivan PF, Brennan JR, McMillan L, Pardo-Manuel de Villena F, 2016 The mouse universal genotyping array: from substrains to subspecies. G3 Genes∣Genomes∣Genetics 6 (2), 263–279. 10.1534/g3.115.022087 PMC4751547 26684931

[R43] Most J, Tosti V, Redman LM, Fontana L, 2017. Calorie restriction in humans: an update. Ageing Res. Rev 39, 36–45. 27544442 10.1016/j.arr.2016.08.005 PMC5315691

[R44] Netzahualcoyotzi C, Pellerin L, 2020. Neuronal and astroglial monocarboxylate transporters play key but distinct roles in hippocampus-dependent learning and memory formation. Prog. Neurobiol 194, 101888. 32693190 10.1016/j.pneurobio.2020.101888

[R45] Neuner SM, Heuer SE, Huentelman MJ, O’Connell KMS, Kaczorowski CC, 2019. Harnessing genetic complexity to enhance translatability of Alzheimer’s disease mouse models: a path toward precision medicine. Neuron 101 (3), 399–411 e395.. 30595332 10.1016/j.neuron.2018.11.040 PMC6886697

[R46] Neuner SM, Wilmott LA, Hope KA, Hoffmann B, Chong JA, Abramowitz J, Birnbaumer L, O’Connell KM, Tryba AK, Greene AS, Savio Chan C, Kaczorowski CC, 2015. TRPC3 channels critically regulate hippocampal excitability and contextual fear memory. Behav. Brain Res 281, 69–77. 25513972 10.1016/j.bbr.2014.12.018 PMC4677051

[R47] Ouellette AR, Neuner SM, Dumitrescu L, Anderson LC, Gatti DM, Mahoney ER, Bubier JA, Churchill G, Peters L, Huentelman MJ, Herskowitz JH, Yang H-S, Smith AN, Reitz C, Kunkle BW, White CC, De Jager PL, Schneider JA, Bennett DA, Seyfried NT, Chesler EJ, Hadad N, Hohman TJ, Kaczorowski CC, 2020. Cross-species analyses identify Dlgap2 as a regulator of age-related cognitive decline and Alzheimer’s dementia. Cell Rep. 32 (9), 108091. 32877673 10.1016/j.celrep.2020.108091 PMC7502175

[R48] Pak HH, Haws SA, Green CL, Koller M, Lavarias MT, Richardson NE, Yang SE, Dumas SN, Sonsalla M, Bray L, Johnson M, Barnes S, Darley-Usmar V, Zhang J, Yen CE, Denu JM, Lamming DW, 2021. Fasting drives the metabolic, molecular and geroprotective effects of a calorie-restricted diet in mice. Nat. Metab 3 (10), 1327–1341. 34663973 10.1038/s42255-021-00466-9 PMC8544824

[R49] Parikh I, Guo J, Chuang K-H, Zhong Y, Rempe RG, Hoffman JD, Armstrong R, Bauer B, Hartz AMS, Lin A-L, 2016. Caloric restriction preserves memory and reduces anxiety of aging mice with early enhancement of neurovascular functions. Aging (Albany NY) 8 (11), 2814–2826. 27829242 10.18632/aging.101094 PMC5191872

[R50] Park DC, Smith AD, Lautenschlager G, Earles JL, Frieske D, Zwahr M, Gaines CL, 1996. Mediators of long-term memory performance across the life span. Psychol. Aging 11 (4), 621–637. 9000294 10.1037//0882-7974.11.4.621

[R51] Pifferi F, Terrien J, Marchal J, Dal-Pan A, Djelti F, Hardy I, Chahory S, Cordonnier N, Desquilbet L, Hurion M, Zahariev A, Chery I, Zizzari P, Perret M, Epelbaum J, Blanc S, Picq JL, Dhenain M, Aujard F, 2018. Caloric restriction increases lifespan but affects brain integrity in grey mouse lemur primates. Commun. Biol 1, 30. 30271916 10.1038/s42003-018-0024-8 PMC6123706

[R52] Recla JM, Robledo RF, Gatti DM, Bult CJ, Churchill GA, Chesler EJ, 2014. Precise genetic mapping and integrative bioinformatics in diversity outbred mice reveals Hydin as a novel pain gene. Mamm. Genome 25 (5), 211–222. 24700285 10.1007/s00335-014-9508-0 PMC4032469

[R53] Ridge PG, Mukherjee S, Crane PK, Kauwe JSK, Alzheimer’s Disease Genetics C, 2013. Alzheimer’s disease: analyzing the missing heritability. PloS one 8 (11), e79771. 24244562 10.1371/journal.pone.0079771 PMC3820606

[R54] Rogina B, Helfand SL, 2004. Sir2 mediates longevity in the fly through a pathway related to calorie restriction. In: Proceedings of the National Academy of Sciences of the United States of America, 101, pp. 15998–16003. 15520384 10.1073/pnas.0404184101 PMC528752

[R55] Scott T, Das S, Martin C, Stewart T, Williamson D, Stein R, Bhapkar M, Pieper C, Rochon J, Roberts S, 2014. CALERIE II: the effect of 25% calorie restriction over two years on cognitive function (629.7). FASEB J. 28 (S1), 629 627..

[R56] Sun LY, Spong A, Swindell WR, Fang Y, Hill C, Huber JA, Boehm JD, Westbrook R, Salvatori R, Bartke A, 2013. Growth hormone-releasing hormone disruption extends lifespan and regulates response to caloric restriction in mice. eLife 2, e01098. 24175087 10.7554/eLife.01098 PMC3810783

[R57] Swerdlow RH, 2007. Is aging part of Alzheimer’s disease, or is Alzheimer’s disease part of aging? Neurobiol. Aging 28 (10), 1465–1480. 16876913 10.1016/j.neurobiolaging.2006.06.021

[R58] Tucker-Drob EM, Briley DA, Harden KP, 2013. Genetic and environmental influences on cognition across development and context. Curr. Dir. Psychol. Sci 22 (5), 349–355. 24799770 10.1177/0963721413485087 PMC4006996

[R59] Tuttle AH, Philip VM, Chesler EJ, Mogil JS, 2018. Comparing phenotypic variation between inbred and outbred mice. Nat. Methods 15 (12), 994–996. 30504873 10.1038/s41592-018-0224-7 PMC6518396

[R60] van Geldorp B, Heringa SM, van den Berg E, Olde Rikkert MG, Biessels GJ, Kessels RP, 2015. Working memory binding and episodic memory formation in aging, mild cognitive impairment, and Alzheimer’s dementia. J. Clinl Exp. Neuropsychol 37 (5), 538–548. 10.1080/13803395.2015.1037722 26011711

[R61] Wahl D, Solon-Biet SM, Wang QP, Wali JA, Pulpitel T, Clark X, Raubenheimer D, Senior AM, Sinclair DA, Cooney GJ, de Cabo R, Cogger VC, Simpson SJ, Le Couteur DG, 2018. Comparing the Effects of low-protein and high-carbohydrate diets and caloric restriction on brain aging in mice. Cell Rep. 25 (8), 2234–2243 e2236.. 30463018 10.1016/j.celrep.2018.10.070 PMC6296764

[R62] Walker CK, Greathouse KM, Boros BD, Poovey EH, Clearman KR, Ramdas R, Muhammad HM, Herskowitz JH, 2021. Dendritic spine remodeling and synaptic tau levels in PS19 tauopathy mice. Neuroscience 455, 195–211. 33346120 10.1016/j.neuroscience.2020.12.006 PMC8142378

[R63] Watts P, Phillips G, Petticrew M, Harden A, Renton A, 2011. The influence of environmental factors on the generalizability of public health research evidence: physical activity as a worked example. Int. J. Behav. Nutr. Phys. Act 8, 128. 22087556 10.1186/1479-5868-8-128 PMC3226640

[R64] Weindruch R, Sohal RS, 1997. Seminars in medicine of the Beth Israel Deaconess Medical Center. Caloric intake and aging. N. Engl. J. Med 337 (14), 986–994. 9309105 10.1056/NEJM199710023371407 PMC2851235

[R65] Weindruch R, Walford RL, Fligiel S, Guthrie D, 1986. The retardation of aging in mice by dietary restriction: longevity, cancer, immunity and lifetime energy intake. J. Nutr 116 (4), 641–654. 3958810 10.1093/jn/116.4.641

[R66] Wilkie SE, Mulvey L, Sands WA, Marcu DE, Carter RN, Morton NM, Hine C, Mitchell JR, Selman C, 2020. Strain-specificity in the hydrogen sulphide signalling network following dietary restriction in recombinant inbred mice. GeroScience 42 (2), 801–812. 32162209 10.1007/s11357-020-00168-2 PMC7205779

[R67] Wimmer ME, Hernandez PJ, Blackwell J, Abel T, 2012. Aging impairs hippocampus-dependent long-term memory for object location in mice. Neurobiol. Aging 33 (9), 2220–2224. 21872364 10.1016/j.neurobiolaging.2011.07.007 PMC3227775

[R68] Witte AV, Fobker M, Gellner R, Knecht S, Flöel A, 2009. Caloric restriction improves memory in elderly humans. In: Proceedings of the National Academy of Sciences of the United States of America, 106, pp. 1255–1260. 19171901 10.1073/pnas.0808587106 PMC2633586

[R69] Wu A, Sun X, Liu Y, 2003. Effects of caloric restriction on cognition and behavior in developing mice. Neurosci. Lett 339 (2), 166–168. 12614920 10.1016/s0304-3940(03)00008-9

[R70] Yu X, Zhang R, Wei C, Gao Y, Yu Y, Wang L, Jiang J, Zhang X, Li J, Chen X, 2021. MCT2 overexpression promotes recovery of cognitive function by increasing mitochondrial biogenesis in a rat model of stroke. Anim. Cells Syst 25 (2), 93–101. 10.1080/19768354.2021.1915379 PMC8118516 34234890

[R71] Zajitschek F, Zajitschek SR, Friberg U, Maklakov AA., 2013. Interactive effects of sex, social environment, dietary restriction, and methionine on survival and reproduction in fruit flies. Age (Dordrecht, Netherlands) 35 (4), 1193–1204. 10.1007/s11357-012-9445-3 PMC3705097 22798158

